# Stealth polymer coatings of reactive oxygen species scavenging nanoparticles for immune response mitigation

**DOI:** 10.1002/btm2.70115

**Published:** 2026-01-27

**Authors:** Jordyn M. Wyse, Monica Prieto Nieto, Jinmin Zhang, Chia George Hsu, Marissa E. Wechsler

**Affiliations:** ^1^ Department of Biomedical Engineering and Chemical Engineering The University of Texas at San Antonio San Antonio Texas USA; ^2^ Department of Kinesiology The University of Texas at San Antonio San Antonio Texas USA; ^3^ Department of Molecular Microbiology and Immunology The University of Texas at San Antonio San Antonio Texas USA

**Keywords:** biomaterials, drug delivery, immunology, nanoparticles, stealth polymers

## Abstract

Elevated levels of reactive oxygen species play an integral role in chronic inflammation. Current treatments for chronic inflammation often ignore reactive oxygen species and instead focus on symptom control or immunosuppression. However, by controlling reactive oxygen species in inflammatory environments, cyclic inflammation can be reduced. Combining reactive oxygen species scavenging delivery systems with stealth coatings can help avoid the innate immune system and enable targeted delivery to sites of inflammation without causing further oxidative stress. For this purpose, poly(propylene sulfide) nanoparticles were synthesized utilizing two different surfactants, Pluronic F‐127 and sucrose monolaurate, adding stealth properties to the coatings of the reactive oxygen species scavenging nanoparticles. Characterization of the nanoparticles demonstrated the surfactant coatings did not affect the scavenging abilities nor the cytocompatibility of the materials. Degradation of the nanoparticles related to the sulfide groups and disulfide bond interactions with reactive oxygen species was also analyzed. Moreover, proinflammatory cytokine secretion from macrophages exposed to the nanoparticles was investigated to determine immune response evasion. Results obtained showed little to no activation of macrophages exposed to nanoparticle formulations in regard to MCP‐1 cytokine release. However, there is room for improvement using glycerol‐based coatings with regard to protecting cells from reactive oxygen species exposure and reducing macrophage activation in relation to IL‐6 and TNF‐alpha. Overall, the nanoparticles investigated have the capabilities to improve inflammatory disease treatments by not only targeting delivery of therapeutics to the site of inflammation, but also avoiding excess immune response recruitment due to incorporation of stealth coatings.

## Graphical abstract

ROS‐responsive nanoparticles were synthesized with various stealth coatings to allow for a mitigated immune response.

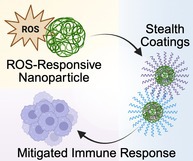

**Translational Impact Statement**

Chronic inflammatory diseases are the main cause of death worldwide. By creating a drug delivery system that is able to interact with the inflammatory environment, while simultaneously reducing inflammation and avoiding an additional immune response, treatments for chronic inflammatory diseases could be improved, lessening the strain on patients and the healthcare system as a whole. For this purpose, reactive oxygen species scavenging nanoparticles with stealth coatings were synthesized to investigate their effect on immune cells.

## 1 INTRODUCTION

Chronic inflammation plays an important role in many different diseases, such as cardiovascular and degenerative diseases, attributing three out of five deaths worldwide.^1^ In a healthy state, a base level of inflammation is present that is beneficial to respond to foreign pathogens and cell death that allows for the removal of hazardous components. However, in many disease states, chronic inflammation can occur which is often characterized by an inflammatory environment that involves the influx of proinflammatory cells and cytokines.^2^ Specifically, an increase in reactive oxygen species (ROS) is frequently found in these proinflammatory environments. ROS are chemical compounds that play a vital role in redox reactions that take place in the body. However, at higher levels over 300 μM, they lead to oxidative stress which causes cell damage, further inducing an immune response, leading to an endless cycle of inflammation.^3^ One of the most common ROS found in the body is hydrogen peroxide^4^ which results from cell metabolism and other redox reactions that occur at the cellular level.^5^ ROS can be located intra‐ or extra‐cellularly, both of which can cause problems for cell function at high levels. Specifically, extracellular ROS, which can be caused by macrophages and other immune cells during inflammation, can lead to the damage of otherwise healthy cells leading to additional inflammation and a continuing cascade.^3^, ^6^ Broadly, high levels of these ROS extracellularly across a variety of diseases further agitate the disease state, and thus, a method of reducing oxidative stress (to modulate inflammation) would be beneficial to controlling inflammation and potentially returning cell/tissue function following inflammatory disease progression.

There are a variety of disease applications that could be improved by ROS scavenging. In vascular diseases, such as atherosclerosis, the inflammatory environment around plaque buildup leads to an increase in ROS which can cause cell stress and death in the endothelial tissue, further damaging the vasculature.^7^, ^8^, ^9^, ^10^ Another case of the role ROS play in disease is related to multiple sclerosis (MS), a neurodegenerative autoimmune disease. MS is characterized by autoreactive immune cells making their way through the blood brain barrier (BBB) and attacking oligodendrocytes and the myelin sheath around neurons.^11^, ^12^, ^13^ This affects neuronal signaling and leads to a progressive reduction in motion and control over the lifetime of the patient. Current MS treatments, such as IFN‐β, focus on slowing disease progression in relapsing–remitting MS, but a method to reverse the damage caused to the nervous system is not currently known.^13^, ^14^ The applications of targeting strategies to reduce ROS for MS patients could decrease the inflammation observed around the oligodendrocytes and thus allow for the possible restoration of the myelin sheath. Overall, the possible effects and applications of ROS scavenging are wide reaching and would provide a new method for the treatment of these chronic inflammatory diseases.

Treatments for chronic inflammation rely on the effective delivery of therapeutics. The main hurdles associated with the direct administration of drugs are inefficiencies in the payload delivered, low circulation time of medication, and reduced patient compliance with a treatment regime. Direct administration often requires multiple drug doses to ensure the therapeutic level of the drug remains constant and effective. To combat this, drug delivery systems have been employed to protect the drug, increase circulation time, reduce off‐target effects, and allow for controlled, targeted delivery. Use of systems derived from biomaterials, such as polymers, has been shown to help overcome challenges associated with traditional drug delivery approaches due to their high tunability and improved functionality. Environmentally responsive polymers add a new facet to their tunability, providing manipulation of the materials and the surrounding environment. Factors such as temperature, pH, magnetism, and others can affect the polymeric system and aid in targeting and delivery.^15^, ^16^ In relation to inflammation, polymers that are capable of responding to ROS have been synthesized. There are a range of ROS‐sensitive polymers that incorporate sulfurs, selenium, tellurium, or boric acid groups.^17^, ^18^, ^19^ They show promise for interacting with ROS, but depending on their structure, can be difficult to incorporate into a functional nanoparticle system that will not buildup in the body. Sulfur‐based polymers for ROS scavenging are the most common as they have proven to be safe and effective in vitro.^20^, ^21^, ^22^ Poly(propylene sulfide) has been shown to interact with reactive oxygen species through sulfide groups present in its structure.^23^, ^24^, ^25^, ^26^ As the sulfide groups of the polymer interact with ROS, oxygen is incorporated causing sulfide groups to change to sulfoxides, and then sulfones.^27^, ^28^ During this transition, the polymer becomes more hydrophilic, leading to degradation and drug release in the body.

While biomaterials serve as useful carriers for therapeutics, there is also an issue of opsonization of materials, which leads to quick removal from circulation, reducing the positive effects of having a delivery system in the first place. To combat this, polymers which avoid opsonization, and thus evade the immune system, can be employed. “Stealth” polymer coatings are used to evade detection by the immune system during intravenous drug delivery so that the circulation times of therapeutics within the body can be increased, allowing for transportation to the targeted site and delivery of the full payload. These stealth polymers work by avoiding opsonization mostly due to steric hindrance. One of the most well‐known and commonly used stealth polymers is poly(ethylene glycol) or PEG.^29^ Use of PEG has been shown to avoid macrophage phagocytosis by reducing protein adsorption. This functionality extends to the variety of PEG‐containing polymers such as Pluronic F‐127, a surfactant useful in the formation of nanoparticle emulsions. However, recent studies have shown that due to the prevalence of PEG in pharmaceuticals, there has been an increase in the population of people who have developed a hypersensitive immune response to PEG itself.^30^ Because of this, there have been efforts to study other possible stealth polymers that could replace PEG and avoid that adaptive immune response. Incorporation of glycerol groups has also been shown to provide stealth capabilities in polymers as glycerol mimics sugar groups naturally found in the body thus, reducing the possibility of opsonization in the bloodstream and subsequent detection by macrophages.^31^ Sucrose monolaurate is a surfactant that is glycerol based, allowing it to be both biocompatible and evade detection of the immune system. By replacing PEG‐based stealth coatings with a glycerol‐based coating, the polymer will retain stealth capabilities without risking the adaptive response that comes along with frequent PEG use.

For these reasons, there is a need to investigate the relationship between polymeric systems and inflammation to pioneer a delivery system capable of providing targeted delivery to locations of inflammation and avoid off‐target effects. Developing a system which focuses on scavenging reactive oxygen species, while reducing the immune cascade, would allow for the treatment of chronic inflammation capable of modulating and reversing disease states. For this purpose, different stealth‐coated poly(propylene sulfide) nanoparticles were synthesized and investigated for their functionality, combining the ROS scavenging nature of the sulfur‐based polymer with the immune evasion techniques of stealth polymers. Synthesizing a polymeric system that is responsive to the local environment, capable of scavenging, reducing ROS, and delivering therapeutics to target sites, while avoiding the immune response will allow for continuous development of multifaceted treatment methods for inflammatory diseases.

## 2 RESULTS

### 2.1 Synthesis and characterization of nanoparticles

Poly(propylene sulfide) (PPS) nanoparticles were synthesized with two separate surfactants at various concentrations (Pluronic F‐127 at 1% w/v and sucrose monolaurate at 4% w/v) to achieve nanoparticles similar in hydrodynamic diameter and surface charge. Surfactant concentration was optimized to reach these matching characteristics (Table S1). The two formulations, poly(propylene sulfide) with a Pluronic F‐127 coating (PPS‐Pluronic) and poly(propylene sulfide) with a sucrose monolaurate coating (PPS‐SM), were similar in hydrodynamic diameter and zeta potential in water and 0.1× PBS (Table 1). However, in 0.1× PBS, PPS‐Pluronic and PPS‐SM resulted in an increase in hydrodynamic diameter and a reduction in zeta potential. No difference in properties was observed between formulations, which indicated that changing the surfactant from Pluronic F‐127 to sucrose monolaurate did not alter the basic characteristics of the synthesized nanoparticles. Fourier transform infrared spectroscopy (FT‐IR) was used to identify nanoparticle composition and surfactant incorporation (Figure S1).

**TABLE 1:** Hydrodynamic diameter and surface charge of the two different PPS formulations of nanoparticles.

Formulation	*D*_*h*_ (nm)	PDI	ζ (mV)
PPS‐Pluronic F‐127 (1% w/v)			
In water	173.10 ± 21.89	0.152 ± 0.043	−22.32 ± 4.74
In 0.1× PBS	221.50 ± 18.65	0.118 ± 0.056	−11.83 ± 1.95
PPS‐Sucrose monolaurate (4% w/v)			
In water	187.74 ± 32.92	0.052 ± 0.020	−23.48 ± 3.26
In 0.1× PBS	255.76 ± 24.91	0.137 ± 0.093	−20.77 ± 8.60

*Note*: Data are reported as mean (*n* = 9) ± standard deviation.

### 2.2 Reactive oxygen species responsiveness and degradation

**FIGURE 1:**
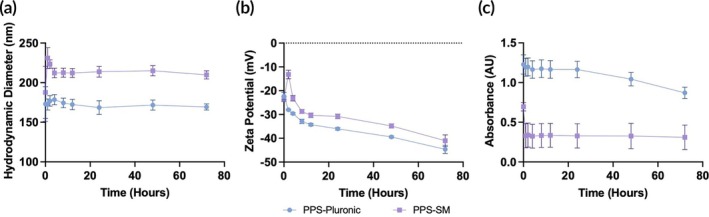
PPS nanoparticle formulations incubated with 5% hydrogen peroxide at 25°C over 72 h. (a) Hydrodynamic diameter of the nanoparticles. (b) Zeta potential of the nanoparticles. (c) Turbidity measured as a function of optical density. Data reported as mean (*n* = 9) ± SE of the mean.

After baseline characterization in solution, nanoparticle characteristics (hydrodynamic diameter, turbidity, and zeta potential) were tested in 5% hydrogen peroxide to investigate and compare the reactive oxygen species scavenging abilities of the two nanoparticle formulations, as well as to controls, nanoparticles suspended in water (Figures 1 and S2). These results showcased decreasing hydrodynamic diameter, turbidity, and surface charge in 5% hydrogen peroxide while those same changes were not observed when nanoparticles were suspended in water. For both nanoparticle formulations in 5% hydrogen peroxide, a slight decrease in hydrodynamic diameter was observed across the 72 h timeframe (Figure 1a). In regards to zeta potential for both nanoparticle formulations, a significant decrease (*p* < 0.0001) between 0 and 72 h was observed (Figure 1b). The main difference between the two formulations in 5% hydrogen peroxide was that while the PPS‐Pluronic nanoparticles slowly decreased in turbidity over time, the PPS‐SM nanoparticles responded with a quick change in turbidity that then plateaued over time (Figure 1c). This indicated an earlier time‐dependent interaction of PPS‐SM nanoparticles with the reactive oxygen species compared to PPS‐Pluronic nanoparticles. This could be beneficial due to the fact that the PPS‐SM nanoparticles may be able to respond more quickly in an inflammatory environment, acting with an increased rate to both scavenge ROS as well as deliver additional therapeutics.

**FIGURE 2:**
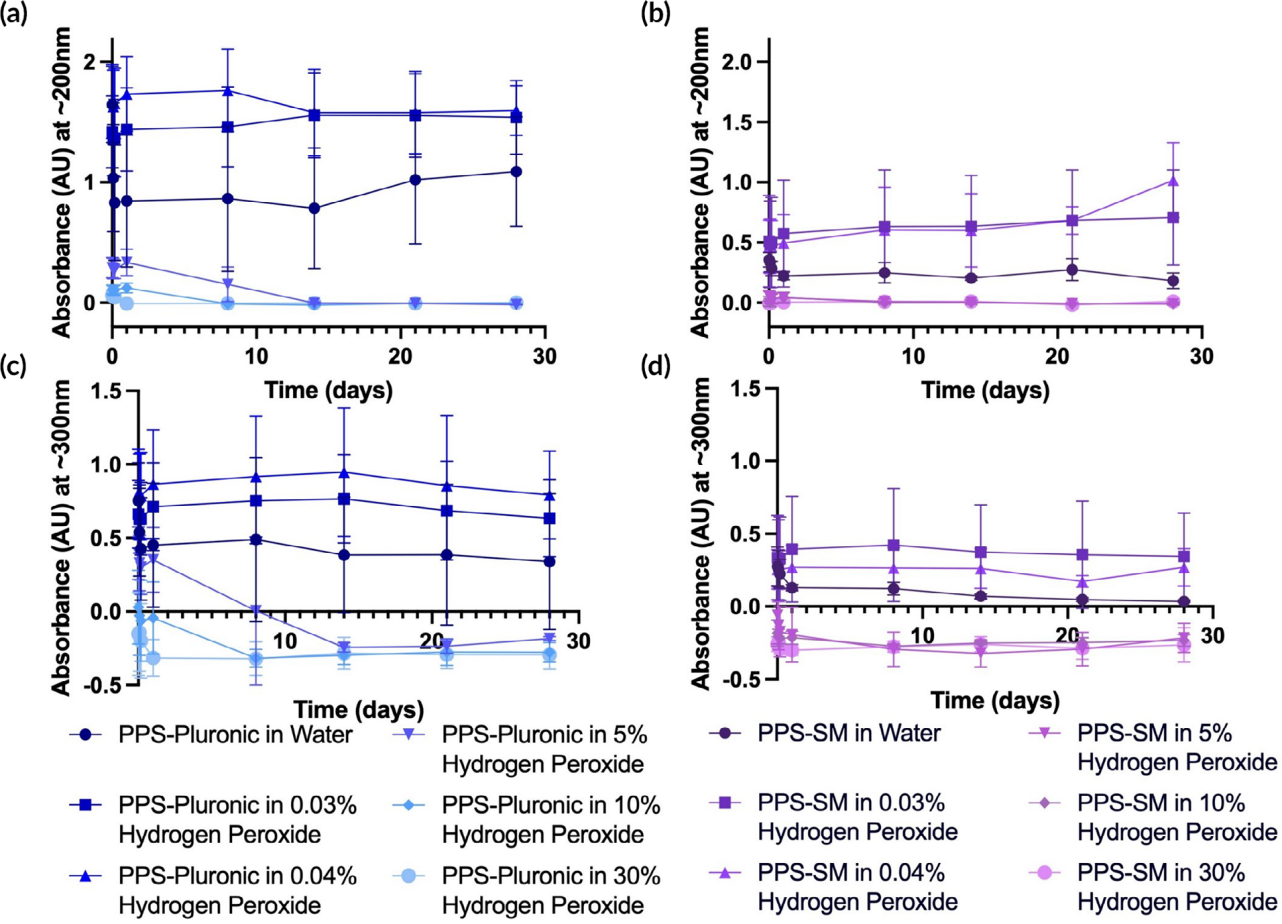
Nanoparticle response to hydrogen peroxide exposure over time via absorbance. (a) PPS‐Pluronic nanoparticles absorption over time at ~200 nm. (b) PPS‐Sucrose monolaurate nanoparticles absorption over time at ~200 nm. (c) PPS‐Pluronic nanoparticles absorption over time at ~300 nm. (d) PPS‐Sucrose monolaurate nanoparticles absorption over time at ~300 nm. Data reported as mean (*n* = 9) ± SE of the mean.

The degradation of the nanoparticles in different concentrations of hydrogen peroxide were cataloged over time by analyzing the UV–vis spectra and subsequent absorbance peaks corresponding to functional groups within nanoparticles and ROS. Specifically, absorbance peaks near ~200 and ~300 nm were monitored as wavelengths corresponding to the sulfide groups within nanoparticles and ROS, respectively^32^, ^33^, ^34^, ^35^ (Table S2). As seen in Figure 2, at ~200 nm, a wavelength that corresponds to the sulfide groups present in the nanoparticles, the change of the sulfide to groups such as sulfoxides due to hydrogen peroxide exposure was observed (Figure 2a,b). At high concentrations of hydrogen peroxide, the peak at ~200 nm was nonexistent, indicating that the nanoparticles rapidly interacted with the hydrogen peroxide and the sulfides quickly degraded. There was also a clear difference in peak absorbance at ~300 nm for the nanoparticles in water compared to the nanoparticles in high concentrations of hydrogen peroxide across both nanoparticle formulations (Figure 2c,d). Given the sharp decline to a negative absorbance in high levels of hydrogen peroxide, it is possible that the absorbance peak, or lack thereof, under those conditions correlated to the presence of reactive oxygen species in the solution, indicating that hydrogen peroxide on its own had an absorbance peak at ~309 nm, however, in the presence of nanoparticles, the absorbance peak was reduced (Figure 2c,d). Overall, both PPS‐Pluronic and PPS‐SM demonstrated similar behaviors when interacting with hydrogen peroxide over time in which their peaks collapsed with exposure to high concentrations of hydrogen peroxide. However, there was a noticeable difference between the two nanoparticle formulations in which the PPS‐Pluronic resulted in a higher overall absorbance compared to the PPS‐SM, indicating that it may require more ROS to be affected.

### 2.3 Cellular compatibility

**FIGURE 3:**
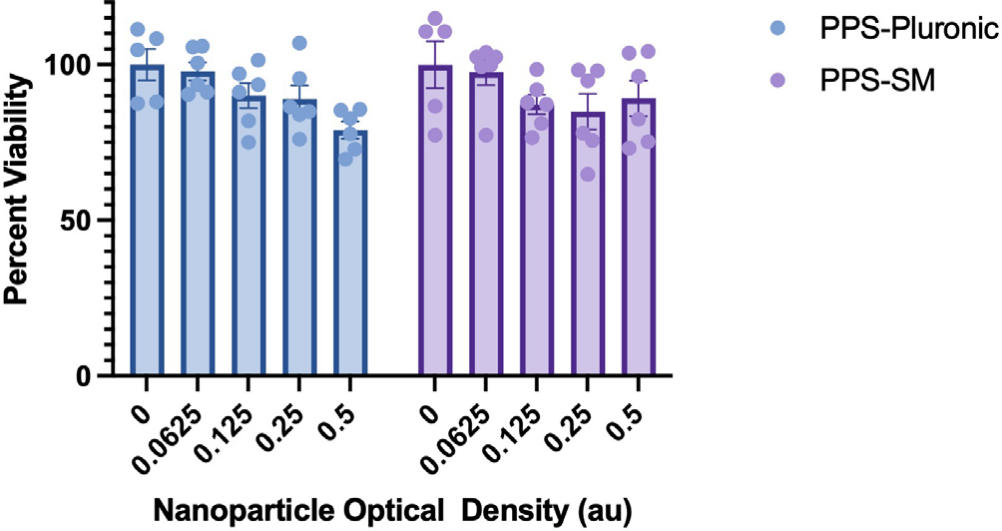
Cytotoxicity evaluation of the PPS nanoparticle formulations against L929 murine fibroblasts. Concentrations of particles were determined optimal at cell viability of 80% or above. Cells exposed to nanoparticles were compared to controls, cells proliferating in media without exposure to nanoparticles as well as a lysis control (not shown, and <5% viable). Data are represented as mean (*n* = 6) ± SE of the mean.

**FIGURE 4:**
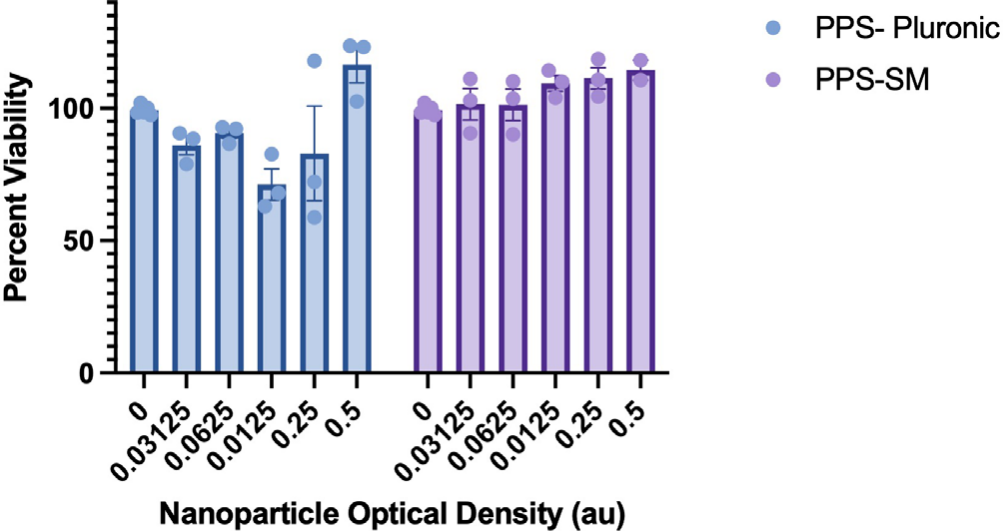
Cytotoxicity evaluation of the PPS nanoparticle formulations against bone marrow‐derived macrophages. Concentrations of particles were determined optimal at cell viability of 80% or above. Cells exposed to nanoparticles were compared to controls, cells proliferating in media without exposure to nanoparticles as well as a lysis control (not shown, and <5% viable). Data are represented as mean (*n* = 3) ± SE of the mean.

It was found that the nanoparticles of both formulations were cytocompatible with fibroblasts, averaging viability over 80% across the nanoparticle concentration gradients tested (Figure 3). No significant difference in viability between formulations or concentrations of nanoparticles was observed (Figure 3). Similar cytocompatibility results were also obtained when primary bone marrow‐derived macrophages were exposed to nanoparticle formulations. Primary bone marrow‐derived macrophages exposed to PPS‐SM nanoparticles resulted in an increasing trend in viability with increasing particle concentration; however, no difference in cell viability was observed between the two formulations or between the concentrations of nanoparticles tested (Figure 4).

### 2.4 ROS exposure of fibroblasts and nanoparticle protecting

**FIGURE 5:**
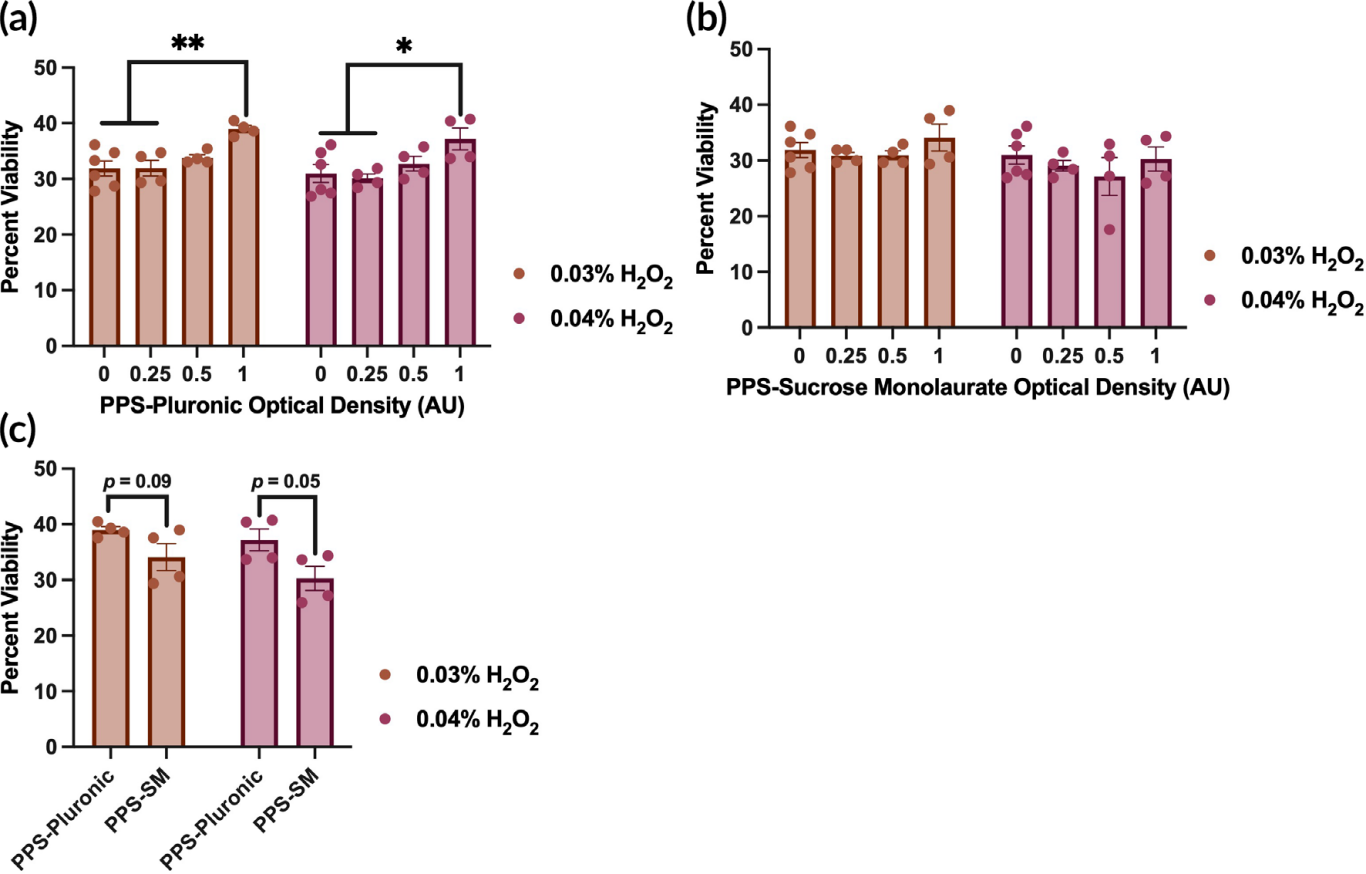
Cell viability after exposure to hydrogen peroxide and PPS nanoparticle formulations. (a) Fibroblasts exposed to PPS‐Pluronic nanoparticles with different concentrations of hydrogen peroxide, *p* < 0.01 between optical densities of nanoparticles with the 0.03% hydrogen peroxide exposure, *p* < 0.05 between optical densities of nanoparticles with the 0.04% hydrogen peroxide exposure. (b) Fibroblasts exposed to PPS‐Sucrose monolaurate (PPS‐SM) nanoparticles with different concentrations of hydrogen peroxide, no significant difference between groups. (c) Comparison of PPS nanoparticle formulations at 1 au. Data are represented as mean (*n* = 4) ± SE of the mean.

When fibroblasts were exposed to hydrogen peroxide in media, cell viability decreased dramatically. However, when the hydrogen peroxide exposed cells were cultured in the presence of PPS nanoparticles, viability increased (Figure 5). Overall, cells exposed to higher concentrations of nanoparticles (1 au) were able to protect the cells from hydrogen peroxide exposure compared to lower nanoparticle concentrations (0.25 au). As seen in Figure 5a, the PPS‐Pluronic formulation of nanoparticles at an optical density of 1 au provided a statistically significant increase in cell viability for both hydrogen peroxide concentrations tested, indicating a protective effect on the cells. While the PPS‐sucrose monolaurate nanoparticles also demonstrated an increase in viability (Figure 5b), it was not statistically significant. A difference in functionality between PPS‐Pluronic and PPS‐SM nanoparticle formulations was observed, as the PPS‐Pluronic increased cell viability compared to PPS‐SM (Figure 5c). These results indicate that higher concentrations of nanoparticles, specifically for the PPS‐sucrose monolaurate, may be necessary to achieve the desired effects of improved cell viability upon exposure to hydrogen peroxide.

### 2.5 Macrophage activation

**FIGURE 6:**
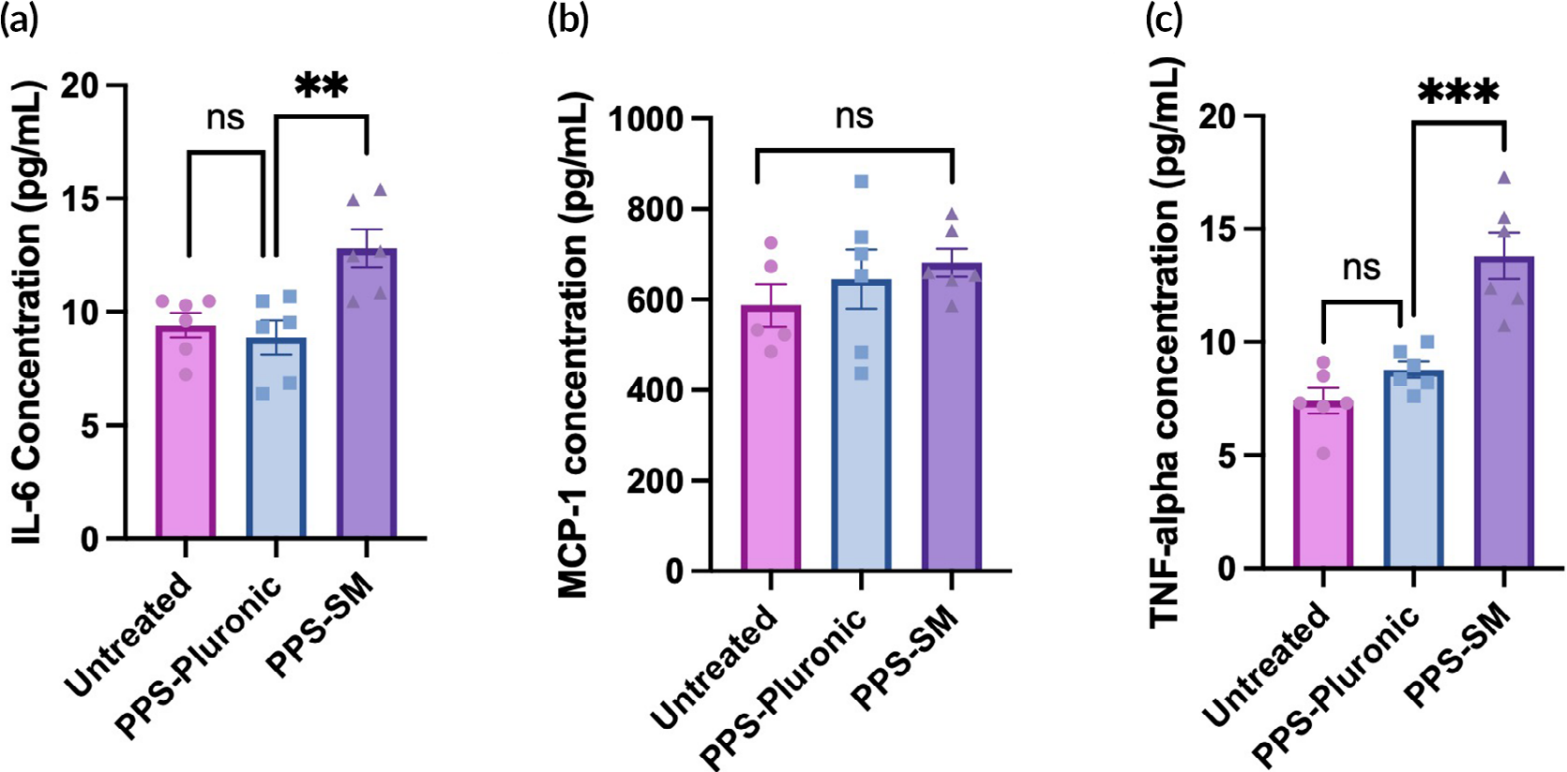
Pro‐inflammatory cytokine release from bone marrow‐derived macrophages that were either untreated, or treated with PPS‐Pluronic nanoparticles, or PPS‐SM nanoparticles for 3 h. (a) IL‐6 release, *p* < 0.01 between nanoparticle formulations. (b) MCP‐1 release, *p* > 0.05 between nanoparticle formulations. (c) TNF‐alpha release, *p* < 0.001 between nanoparticle formulations. Data are represented as mean (*n* = 6) ± SE of the mean.

The effect of nanoparticle exposure on the classical activation of macrophages to the M1 phenotype was tested via pro‐inflammatory cytokine release. Specifically, release of IL‐6, MCP‐1, and TNF‐alpha was investigated in macrophages that were untreated (not exposed to nanoparticles) and exposed to nanoparticles. Minimal cytokine release was seen from the macrophages exposed to the nanoparticles, comparable to the low levels seen released from the untreated macrophages, indicating that the nanoparticles did not cause high levels of activation in the cells (Figure 6). When macrophages were exposed to PPS‐Pluronic nanoparticles, no difference in cytokine secretion was observed between untreated cells and those that were exposed to the nanoparticles across all cytokines tested. However, a significant difference in cytokine release of IL‐6 (*p* < 0.01) and TNF‐alpha (*p* < 0.001) was observed from cells exposed to PPS‐SM compared to untreated macrophages. Related to this, a significant difference in cytokine release from the macrophages exposed to the two different nanoparticle formulations was observed (Figure 6). Across all studies, there was a significant difference in cytokine release from cells exposed to nanoparticles compared to cells exposed to lipopolysaccharide (LPS) (Figure S3). The cytokine release from macrophages exposed to LPS demonstrated a profile of classically activated macrophages and demonstrated macrophages exposed to nanoparticles were not activated. In addition, the cytokine release profiles obtained demonstrated that while the sucrose monolaurate coating was effective at minimizing macrophage activation compared to the high levels caused by the LPS, the Pluronic F‐127 coating resulted in the lowest macrophage activation by cytokine secretion, with results similar to cells untreated and not exposed to nanoparticles.

## 3 DISCUSSION

Chronic inflammation is the leading cause of disease worldwide and thus requires effective treatments for management and resolution. While there are a wide variety of treatments on the market, there is a need for improved efficacy which can be carried out using biomaterials, such as polymeric nanoparticles. Current treatments of inflammatory diseases often focus on either mitigating symptoms, as in the case of atherosclerosis, where the goal is to reduce cholesterol which can lead to plaque buildup, or to tamp down the immune response as a whole, as in multiple sclerosis, which places patients at risk for the development of infection due to being immunocompromised.^7^, ^8^, ^13^, ^14^, ^36^, ^37^, ^38^ The use of nanoparticles as delivery systems provides the ability to control release and target delivery to specific sites in the body for the aforementioned disease treatments. More specifically, nanoparticles allow for the targeting of specific chronic inflammation environmental factors which can overcome the hurdles of the traditional systemic immunocompromising effects. Environmentally responsive nanoparticles can be tuned specifically to react to high levels of reactive oxygen species that occur during inflammation, thus targeting areas of high inflammation. This ROS scavenging capability enables reduction of the inflammatory environment without stopping the immune system altogether.^39^ Another benefit of polymeric biomaterials is the ability to utilize stealth coatings to reduce the immune response caused by identification of foreign bodies. This allows the nanoparticles to effectively deliver their therapeutic load to sites of inflammation without causing further unnecessary flare‐ups.

The nanoparticle synthesis carried out in this study took advantage of the ROS‐responsive nature of poly(propylene sulfide) combined with a variety of stealth coatings to reduce issues with inflammation caused specifically by the biomaterial carrier. The synthesis scheme used allowed for fabrication of nanoparticles similar in their characteristics using two different stealth coating surfactants. The general structure of the nanoparticles contained a poly(propylene sulfide) core with a surfactant coating providing stealth properties to reduce interactions with the innate immune system. By optimizing the concentration of surfactant used in material syntheses, both nanoparticle formulations (PPS‐Pluronic and PPS‐SM) resulted in similar hydrodynamic diameters and zeta potentials allowing for them to be directly compared across studies. Their nanoscale dimensions make these particles well‐suited for extracellular interactions at sites of inflammation.^40^, ^41^ Intravenous delivery of these materials would be optimal for the treatment of inflammation in the vasculature, either along sites of plaque buildup or leaky endothelial barriers from immune cell migration.^5^, ^9^, ^12^, ^42^, ^43^ Further characterization of the nanoparticles focused on the interactions between the materials and reactive oxygen species. More specifically, the nanoparticles were exposed to hydrogen peroxide which is one of the leading reactive oxygen species in the body.^2^, ^3^, ^4^, ^6^ Interactions of nanoparticles with reactive oxygen species caused the poly(propylene sulfide) to undergo chemical changes. Specifically, the sulfide group can turn into a sulfoxide, and then a sulfone as oxygen becomes incorporated into the material.^27^, ^28^ During this process, the nanoparticle characteristics change; the materials become more hydrophilic allowing them to be more easily degraded in the body via hydrolysis, as well as enzymatic breakdown of the disulfide crosslinks. This, in turn, allows for improved therapeutic delivery at sites of high reactive oxygen species concentrations due to increased degradation of the carrier and release of the drug. This property has been previously utilized in PPS drug delivery carriers as part of a block co‐polymer where when ROS were encountered, led to the dissolution of hydrogels.^44^ Both nanoparticle formulations synthesized in this work led to a decrease in hydrodynamic diameter, zeta potential, and turbidity (as a function of absorbance) upon exposure to ROS. These results are similar to a previous study using poly(propylene sulfide) in which the material also demonstrated a decrease in turbidity in association with ROS exposure.^24^ This was further supported by our UV–vis degradation study which demonstrated the interaction of the nanoparticles with hydrogen peroxide, where higher concentrations of hydrogen peroxide (>5% v/v) led to a decrease in absorbance peaks compared to the nanoparticles in water alone. The proposed mechanism of degradation of the nanoparticles is by way of ROS interacting with, and breaking apart, the disulfide bonds crosslinking the nanoparticles, thus causing the nanoparticles to disassociate.^45^, ^46^ Another part of the degradation mechanism is the interaction of the nanoparticle with ROS causing the sulfide groups to become sulfoxides, which in turn increases the hydrophilicity of the nanoparticles and subsequent breakdown over time within the body.^24^, ^45^, ^46^ Overall, this significant difference in absorbance observed over time across different concentrations of reactive oxygen species indicated that the addition of stealth coatings did not reduce the ability of the polypropylene sulfide to interact with the reactive oxygen species, allowing for greater application of this work.

After characterization demonstrated that both nanoparticle formulations were similar and viable for their intended application, the nanoparticles were then tested against cells (fibroblasts and macrophages) to determine cytocompatibility. Nanoparticles were tested at a range of concentrations determined by their optical density for cytocompatibility. It was observed that L929 murine fibroblasts remained viable across all concentrations and formulations of nanoparticles tested, indicating that the nanoparticles were cytocompatible and can be used for further in vitro studies. Further in vitro studies using fibroblasts were used to investigate the ability of the nanoparticles to protect cells in the event of exposure to high levels of hydrogen peroxide (ROS). Results demonstrated a statistically significant improvement in cell viability when higher concentrations of PPS‐Pluronic nanoparticles (1 au) were applied upon exposure to hydrogen peroxide. This indicated that higher concentrations of nanoparticles would be ideal moving forward to ensure ROS scavenging at beneficial levels. Next, cytocompatibility testing was carried out with primary bone marrow‐derived macrophages. The bone marrow‐derived macrophages were used due to their function as innate immune cells and one of the first lines of defense in inflammatory diseases. Specific use of primary monocytes differentiated into macrophages enables translation to future in vivo work applied to inflammatory diseases. Cytocompatibility tests with these macrophages showcased viability across the nanoparticle concentrations and formulations tested. This further solidified the compatibility of these nanoparticles in vitro and lends itself to future translational work.

Lastly, testing macrophage activation allowed for an understanding of whether the two different stealth coatings on synthesized nanoparticles had the ability to evade macrophages and thus, the innate immune system. The goal was to fabricate and utilize nanoparticles that would not cause further inflammation while trying to deliver therapeutics for inflammatory disease application. This also mirrors previous work using PEG and glycerol‐based stealth polymers that showcase their ability to limit classical macrophage activation.^29^, ^31^ It has also been demonstrated that PEG‐based coatings of polysulfide nanoparticles aid in reducing phagocytic activity and subsequent uptake of the nanoparticles by macrophages.^22^ The current study focused on cytokine secretion, where there was no significant difference between the two nanoparticle formulations in their macrophage secretion of MCP‐1. MCP‐1 is a chemokine focused on macrophage recruitment. Results obtained indicated that both formulations of nanoparticles had limited effects on macrophage classical activation.^47^ On the other hand, a significant difference in IL‐6 and TNF‐alpha secretion was observed upon cell exposure to PPS‐Pluronic versus the PPS‐SM nanoparticles. The macrophages exposed to the PPS‐Pluronic nanoparticles were statically similar to the macrophages not exposed to any nanoparticles, while the PPS‐SM caused a significant difference in IL‐6 and TNF‐alpha release compared to the untreated macrophages. IL‐6 is a cytokine expressed by macrophages when interacting with pathogens or other foreign bodies, while TNF‐alpha is related to inducing cell death and advancing the inflammatory cascade.^48^, ^49^ These release profiles were also compared to macrophages activated by LPS, a protein found in bacterial cell membrane known to activate macrophages. Cytokine release profiles of macrophages exposed to LPS were 10‐fold higher compared to profiles obtained from cells exposed to the nanoparticles. Overall, the secretion of inflammatory cytokines from macrophages exposed to nanoparticles was similar to that of inactivated macrophages, demonstrating the synthesized nanoparticles could be utilized in translational studies as they do not elicit an innate immune response that would counteract the anti‐inflammatory effects of ROS reduction.

Overall, the PPS nanoparticles synthesized with either stealth coating of Pluronic F‐127 or sucrose monolaurate were effective at interacting with extracellular ROS while evading macrophage detection. In addition, the synthesized nanoparticles were comparable to previously investigated ROS sensitive polymers which contain a sulfur component in the backbone along with crosslinked disulfide bonds, allowing the material to interact with ROS in two ways.^24^, ^45^, ^46^ Compared to other ROS sensitive nanoparticles, such as those that contain selenium, sulfur containing systems are more readily synthesized and simply disassociate due to their reversible disulfide bonds.^17^, ^18^, ^19^ However, there is room for improvement using glycerol‐based coatings with regards to protecting cells from ROS and reducing macrophage activation. Future work could focus on engineering and fabricating a synthetic form of the sucrose monolaurate with a longer chain thus, providing greater steric hinderance while maintaining glycerol groups that currently provide stealth properties. This would further reduce chances of opsonization and thus, detection by, and activation of, macrophages. Based on the results obtained, the materials developed hold promise for use in translational applications for treatment of inflammatory diseases through ROS scavenging.

## 4 MATERIALS AND METHODS

### 4.1 Nanoparticle synthesis

#### 4.1.1 Pluronic F‐127 coating: PPS‐Pluronic

Poly(propylene sulfide) (PPS) nanoparticles were synthesized using an anionic ring opening polymerization, adapted from previous studies.^23^, ^24^ The polymer and nanoparticle were synthesized simultaneously in this scheme, with the polymer forming and associating within the emulsion of the synthesis to form nanoparticles. One mL of propylene sulfide (TCI America, Portland, OR) was added to Pluronic F‐127 (1% w/v, Sigma‐Aldrich, St. Louis, MO) in 25 mL of ultrapure water. The reaction was initiated using 27.4 μL of 1,3‐propanedithiol (Thermo Scientific, Waltham, MA) and catalyzed with 152 μL of 1,8‐Diazabicyclo[5.4.0]undec‐7‐ene (DBU) (Thermo Scientific, Waltham, MA). The reaction was carried out at room temperature while stirring for 24 h under inert conditions. After polymerization, the nanoparticles were crosslinked via disulfide bonds by exposure to air for 2 h. Particles were then purified via dialysis using 8 kDa mesh size (Spectra/Por 1, Spectrum Labs, CA) against ultrapure water and stored in solution at 4°C until further use. Synthesis was completed in triplicate.

#### 4.1.2 Sucrose monolaurate coating: PPS‐SM

Poly(propylene sulfide) nanoparticles coated in sucrose monolaurate were synthesized following a similar scheme as the Pluronic F‐127 coated nanoparticles. Briefly, one mL of propylene sulfide was added to sucrose monolaurate (4% w/v, Combi‐Blocks, CA) in 25 mL of ultrapure water, initiated with 27.4 μL 1,3‐propanedithiol, and catalyzed with 152 μL DBU. The nanoparticles were crosslinked via exposure to air for 2 h, purified via dialysis, and stored at 4°C in solution. Synthesis was completed in triplicate.

### 4.2 Nanoparticle characterization

Nanoparticle hydrodynamic diameter and zeta potential were determined using dynamic light scattering (Malvern Zetasizer Nano Series, Malvern, UK). Nanoparticles suspended in either water or 0.1× phosphate buffered saline (PBS) (Fisher Scientific, Waltham, MA) at an optical density of 0.5 were used for measurements of hydrodynamic diameter and surface charge. Nanoparticle composition was confirmed with Fourier transform infrared spectroscopy (FT‐IR) using a Nicolet iS20 FT‐IR Spectrometer (Fisher Scientific, Waltham, MA) with a germanium crystal. Background spectra were collected prior to each sample and used for background subtraction.

### 4.3 Reactive oxygen species responsiveness and degradation

The interaction of reactive oxygen species and the two formulations of poly(propylene sulfide) nanoparticles was determined using hydrogen peroxide. Nanoparticles were incubated with 5% hydrogen peroxide (Fisher Scientific, Waltham, MA) or in ultrapure water alone for 72 h at room temperature while mixing. At selected time points, the hydrodynamic diameter, zeta potential, and turbidity (as a function of absorbance) were determined utilizing dynamic light scattering and UV–vis spectrophotometry (BioTek Epoch 2 Microplate Spectrophotometer, BioTek, Winooski, VT), respectively. Specifically, absorbance of the nanoparticles at 500 nm was determined and used to approximate solution turbidity based on optical density. To track degradation of the nanoparticles and possible reactive oxygen species scavenging, UV–vis spectra of the nanoparticles in hydrogen peroxide were obtained over 28 days. Nanoparticles of both formulations at an optical density of 1 au were incubated in either ultrapure water, 0.03%, 0.04%, 5%, 10%, or 30% hydrogen peroxide (v/v) at room temperature while mixing. At selected time points, the absorbance spectra were obtained utilizing UV–vis spectrophotometry (BioTek Epoch 2 Microplate Spectrophotometer, BioTek, Winooski, VT). The wavelengths of interest were approximately 200 and 300 nm as those corresponded to the possible presence of the sulfide groups and reactive oxygen species, respectively.

### 4.4 Fibroblast compatibility

To ensure cytocompatibility of the nanoparticles, L929 murine fibroblasts (CCl‐1, American Type Culture Collection, VA) were exposed to the two formulations of nanoparticles at different concentrations. Nanoparticles were prepared for cell studies by first centrifuging to pellet, then were sterilized under UV light for 20 min, and washed with sterile 1× PBS. Finally, the nanoparticles were centrifuged, the supernatant was removed, and nanoparticles were resuspended in cell media. Specifically, the media used for these studies was phenol red‐free Dulbecco's modification of Eagle's medium (DMEM, Corning, NY) containing L‐glutamine supplemented with 2% fetal bovine serum (FBS, Corning, NY) and 1% penicillin/streptomycin (Corning, NY). L929 cells were seeded at 10,000 cells/well in a 96 well plate and allowed to attach for 24 h under standard cell culture conditions (humidified, 37°C, 5% CO_2_/95% air environment). Then, the cells were exposed to both formulations of nanoparticles at a gradient of optical densities ranging from 0.03125 to 0.5 au. To compare with the nanoparticle‐exposed cells, cells were exposed to Pierce IP Lysis Buffer (Fisher Scientific, Waltham, MA) (positive control), and cells maintained in normal growth media (negative control). After 24 h of cell exposure to either nanoparticles or positive and negative conditions, the media was removed from the cells, the cells were washed twice with 1× PBS, and a MTS assay (CellTiter 96® AQueous One Solution Cell Proliferation Assay, Promega, WI) was used to determine cell proliferation and viability.

### 4.5 ROS exposure of fibroblasts and nanoparticle protecting

To analyze whether the nanoparticles had an impact on cells exposed to hydrogen peroxide, procedures were modified from fibroblast compatibility studies. Briefly, L929 murine fibroblast cells were seeded at 10,000 cells/well in a 96 well plate in phenol red‐free growth media and allowed to attach for 24 h under standard cell culture conditions (humidified, 37°C, 5% CO_2_/95% air environment). Then the cells were exposed to two different concentrations of hydrogen peroxide in media: 0.03% or 0.04% v/v. These concentrations correlate to high levels of hydrogen peroxide that can be found within inflammatory environments within the body.^50^, ^51^, ^52^ Immediately after hydrogen peroxide exposure, the prepared nanoparticles of both formulations were added at a gradient of optical densities ranging from 0.25 to 1 au. Controls were cells cultured in media and not exposed to nanoparticles, and cells exposed to hydrogen peroxide in media but not protected with nanoparticles. After 3 h of exposure, the media was removed from the cells, the cells were washed twice with 1× PBS, and an MTS assay was used to determine cell proliferation and viability.

### 4.6 Monocyte isolation and macrophage differentiation

Bone marrow cells were harvested from C57BL/6J mice (Jackson Laboratory, stock #000664) using a centrifugation‐based spin‐through technique. After tibiae and femora were dissected and cleared of muscle and connective tissue, the bones were briefly rinsed in ice‐cold, sterile DMEM containing 10% FBS and 1% penicillin/streptomycin. A 0.5 mL microcentrifuge tube was perforated at its base using an 18‐gauge sterile needle, nested inside a 1.5 mL tube, and the bone was inserted vertically into the inner tube. Centrifugation at 100×*g* for 1 min expelled the bone marrow into the outer tube. Marrow from all samples was pooled, resuspended in complete DMEM, and passed through a 70 μm cell strainer (Corning, cat. no. 431751, NY) to remove debris. The resulting cells were pelleted, resuspended in freezing medium (90% FBS and 10% DMSO), and stored either in liquid nitrogen (for long‐term preservation) or at −80 °C (for short‐term use). For macrophage generation, bone‐marrow‐derived mononuclear cells were cultured for 7 days at 37 °C in a humidified 5% CO₂ environment in DMEM supplemented with 10% FBS and 1% penicillin/streptomycin. Monocyte‐to‐macrophage differentiation was induced by supplementing the culture medium with macrophage colony‐stimulating factor (M‐CSF; BioLegend, #576406, CA) at 10 ng/mL. Fresh M‐CSF‐containing medium was added every other day until mature macrophage morphology was observed on day 7.

### 4.7 Macrophage cytocompatibility

Cytocompatibility of the two formulations of nanoparticles was completed with bone marrow‐derived macrophages, following the same procedures as the L929 cytocompatibility study. Briefly, nanoparticles were prepared for cell culture and then suspended in media at a gradient of optical densities ranging from 0.03125 to 0.5 au. The nanoparticles were applied 24 h after seeding the bone marrow‐derived macrophages that were previously differentiated and trypsinized at 5000 cells/well in a 96 well plate. Controls were cells exposed to Pierce IP Lysis Buffer (Fisher Scientific, Waltham, MA) (positive control) and cells maintained in normal growth media (negative control). After 24 h of nanoparticle exposure while the cells were maintained under standard cell culture conditions, the media was removed, the cells were washed with 1× PBS, and the Promega MTS assay was applied to determine cell viability.

### 4.8 Macrophage activation

Bone marrow derived monocytes were cultured over 7 days, following the macrophage differentiation protocol previously described in a 12 well plate. After 7 days when the monocytes had become macrophages, the cells were exposed to both formulations of nanoparticles at an optical density of 0.5 au, determined by the cytocompatibility study. This was compared against cells exposed to LPS (Invitrogen, MA) at 100 ng/mL, as a positive control for macrophage activation, as well as a negative control (untreated macrophages maintained in complete growth media). Exposure was carried out for 3 h while the cells were incubated under standard cell culture conditions. After 3 h, the media from each well was removed and used to test for pro‐inflammatory cytokine release via enzyme‐linked immunosorbent assays (ELISA). Specifically, TNF‐alpha, MCP‐1, and IL‐6 presence was tested using ELISA kits (Biolegend, CA) following instructions provided by the manufacturer. Absorbance was read using a UV–vis spectrophotometer (BioTek, Winooski, VT).

### 4.9 Statistical analyses

All experiments were repeated at least three times. Data are reported as mean ± standard error of the mean. Statistical analyses were performed using GraphPad Prism 10.5 (GraphPad Software, LLC, CA). One way ANOVA with post hoc Tukey tests was conducted for cytokine release studies. Two way ANOVA with post hoc Tukey tests were conducted for cytocompatibility tests as well as hydrogen peroxide cell exposure experiments. Unpaired T tests were used to compare PPS‐Pluronic and PPS‐sucrose monolaurate formulations in hydrogen peroxide exposure experiment. *p* values <0.05 were considered significant.

## 5 CONCLUSIONS

Overall, ROS‐responsive stealth coated nanoparticles were successfully synthesized, characterized, and tested in vitro for immune response activation. By altering the surfactant, and thus, the stealth coating between Pluronic F‐127 or sucrose monolaurate, the nanoparticles were able to avoid classical macrophage activation while retaining their characteristics and scavenging interactions of reactive oxygen species. The nanoparticles synthesized demonstrated compatibility with fibroblasts and macrophages, showcasing their potential in future in vitro and in vivo studies. Regarding macrophage activation, cell exposure to nanoparticles showcased low or no activation of the macrophages, indicating successful stealth properties of materials. While the glycerol‐based sucrose monolaurate coating performed similarly to the PEG‐based Pluronic F‐127 in most experiments, the Pluronic F‐127 coating provided less macrophage activation compared to the sucrose monolaurate coating. These results require further investigation, moving from the innate immune response activation (via the macrophage study) into possible adaptive immune responses. Exploration of this work would further demonstrate the efficacy of sucrose monolaurate‐based coatings as a replacement for current PEG‐based stealth options on the market.

## AUTHOR CONTRIBUTIONS

**Jordyn M. Wyse:** conceptualization; methodology; data collection and analysis; writing ‐ original draft and editing. **Monica Prieto Nieto:** methodology; data collection. **Jinmin Zhang:** methodology; macrophage acquisition. **Chia George Hsu:** methodology; writing ‐ review and editing. **Marissa E. Wechsler:** Conceptualization; funding acquisition; writing – original draft; methodology; writing – review and editing; supervision.

## FUNDING INFORMATION

The authors would like to acknowledge funding support by the National Science Foundation (2137423 to Jordyn M. Wyse) and the University of Texas System Trauma Research and Combat Casualty Care Collaborative (to Marissa E. Wechsler).

## CONFLICT OF INTEREST STATEMENT

The authors have no conflicts of interest to declare.

## ETHICS STATEMENT

This study was conducted in compliance with the Animal Welfare Act and the Implementing Animal Welfare Regulations, in accordance with the principles of the Guide for the Care and Use of Laboratory Animals, and was approved by the Institutional Animal Care and Use Committee at the University of Texas at San Antonio.

## Supporting information


**DATA S1.** Supporting information.

## Data Availability

The data that support the findings of this study are available from the corresponding author upon reasonable request.
