# Supplementary material for: Stealth polymer coatings of reactive oxygen species scavenging nanoparticles for immune response mitigation
Source: Bioeng Transl Med. 2026 Jan 27;11(3):e70115. doi: 10.1002/btm2.70115 (PMC13247422; doi:10.1002/btm2.70115)
Supplement: Supplementary file 1 — DATA S1. Supporting information. [file BTM2-11-e70115-s001.docx]

**Supplementary Information**

*Stealth Polymer Coatings of Reactive Oxygen Species Scavenging Nanoparticles*

*for Immune Response Mitigation*

Jordyn M. Wyse^a^, Monica Prieto Nieto^a^, Jinmin Zhang^b,c^, Chia George Hsu^b,c^, Marissa E. Wechsler^a^

^a^ Department of Biomedical Engineering and Chemical Engineering, The University of Texas at San Antonio, San Antonio, TX, USA

^b^ Department of Kinesiology, The University of Texas at San Antonio, San Antonio, TX, USA

^c^ Department of Molecular Microbiology and Immunology, The University of Texas at San Antonio, San Antonio, TX, USA

Table of Contents:

| **Table S1:** Hydrodynamic diameter and polydispersity index of the PPS-Sucrose monolaurate formulations of nanoparticles | **3** |
| --- | --- |
| **Figure S1:** Normalized offset FT-IR Absorbance Spectra for nanoparticle formulations and surfactants alone | **3** |
| **Table S2:** Peak wavelengths for UV-Vis Spectra degradation study. | **4** |
| **Figure S2:** PPS nanoparticle formulations incubated with 5% hydrogen peroxide or ultrapure water at 25 °C over 72 hours | **4** |
| **Figure S3:** Pro-inflammatory cytokine release from bone marrow-derived macrophages untreated, treated with PPS-Pluronic nanoparticles, PPS-SM nanoparticles, and LPS over 3 hours | **5** |

**Materials and Methods**

*Synthesis*

Poly(propylene sulfide) (PPS) nanoparticles were synthesized using an anionic ring opening polymerization, adapted from previous studies ^1,2^. One mL of propylene sulfide (TCI America, Portland, OR, USA) was added to sucrose monolaurate (Combi-Blocks, CA, USA) at varied percentages in 25 mL of ultrapure water, the reaction was initiated using 27.4 μl of 1,3-propanedithiol (Thermo Scientific, Waltham, MA, USA), and catalyzed with 152 μl of 1,8-Diazabicyclo[5.4.0]undec-7-ene (DBU) (Thermo Scientific, Waltham, MA, USA). The sucrose monolaurate percentages tested were 1%, 2%, and 4% w/v in ultrapure water. The reaction was carried out at room temperature while stirring for 24 hours under inert conditions. After polymerization, the nanoparticles were crosslinked via disulfide bonds by exposure to air for 2 hours. Particles were then purified via dialysis using 8kDa mesh size (Spectra/Por 1, Spectrum Labs, CA, USA) against ultrapure water and stored in solution at 4°C until further use. Synthesis was completed in triplicate.

*Reactive Oxygen Species Responsiveness*

The interaction of reactive oxygen species and the two formulations of poly(propylene sulfide) nanoparticles was determined using hydrogen peroxide. Nanoparticles were incubated with 5% hydrogen peroxide (Fisher Scientific, Waltham, MA, USA) in ultrapure water for 72 hours at room temperature while mixing or in ultrapure water alone. At selected time points, the hydrodynamic diameter, zeta potential, and turbidity (as a function of absorbance) were determined utilizing dynamic light scattering and UV-vis spectrophotometry (BioTek Epoch 2 Microplate Spectrophotometer, BioTek, Winooski, VT, USA), respectively. Specifically, absorbance of the nanoparticles at 500nm was determined and used to approximate solution turbidity based on optical density.

*Macrophage Activation*

Bone marrow-derived monocytes were cultured over 7 days, following the macrophage differentiation protocol in a 12 well plate. After 7 days, when the monocytes had become macrophages, the cells were exposed to both formulations of nanoparticles at an optical density of 0.5 au, determined by the cytocompatibility study. This was compared against cells exposed to LPS (Invitrogen, MA, USA) at 100ng/mL, as a positive control for macrophage activation, as well as a negative control (untreated macrophages maintained in complete growth media). Exposure was carried out for three hours while the cells were incubated under standard cell culture conditions. After three hours, the media from each well was removed and used to test for pro-inflammatory cytokine release via enzyme-linked immunosorbent assays (ELISA). Specifically, TNF-alpha, MCP-1, and IL-6 presence was tested using ELISA kits (Biolegend, CA, USA) following instructions provided by the manufacturer. Absorbance was read using a UV-vis spectrophotometer (BioTek, Winooski, VT, USA).

**Table S1. Hydrodynamic diameter and polydispersity index of the PPS-Sucrose monolaurate formulations of nanoparticles.**

| **Formulation** | **D_h_ (nm)** | **PDI** |
| --- | --- | --- |
| PPS-Sucrose monolaurate (1% w/v) | 298.57 ± 1.81 | 0.0128 ± 0.012 |
|  |  |  |
| PPS-Sucrose monolaurate (2% w/v) | 229.33 ± 4.11 | 0.039 ± 0.023 |
|  |  |  |
| PPS-Sucrose monolaurate (4% w/v) | 173.10 ± 7.29 | 0.152 ± 0.014 |
|  |  |  |

Data are reported as mean (n=9) ± standard error of the mean.

**Figure S1. Normalized offset FT-IR absorbance spectra for nanoparticle formulations and surfactants alone.** All spectra were normalized to the maximum absorbance at 1104 cm^-1^ associated with the peak in Pluronic F-127. PPS= poly(propylene sulfide), SM= sucrose monolaurate.

**Table S2. Peak wavelengths for UV-vis spectra degradation study.**

| **Formulation** | **Aqueous Solution** | **Wavelength for Sulfide (nm)** | **Wavelength for ROS (nm)** |
| --- | --- | --- | --- |
| Poly(propylene sulfide)-Pluronic F-127 | Water | 206 | 282 |
|  | 0.03% Hydrogen Peroxide | 206 | 282 |
|  | 0.04% Hydrogen Peroxide | 206 | 282 |
|  | 5% Hydrogen Peroxide | 206 | 282 |
|  | 10% Hydrogen Peroxide | 206 | 294 |
|  | 30% Hydrogen Peroxide | 206 | 309 |
| Poly(propylene sulfide)-Sucrose Monolaurate | Water | 225 | 282 |
|  | 0.03% Hydrogen Peroxide | 225 | 282 |
|  | 0.04% Hydrogen Peroxide | 225 | 282 |
|  | 5% Hydrogen Peroxide | 225 | 282 |
|  | 10% Hydrogen Peroxide | 225 | 294 |
|  | 30% Hydrogen Peroxide | 225 | 309 |


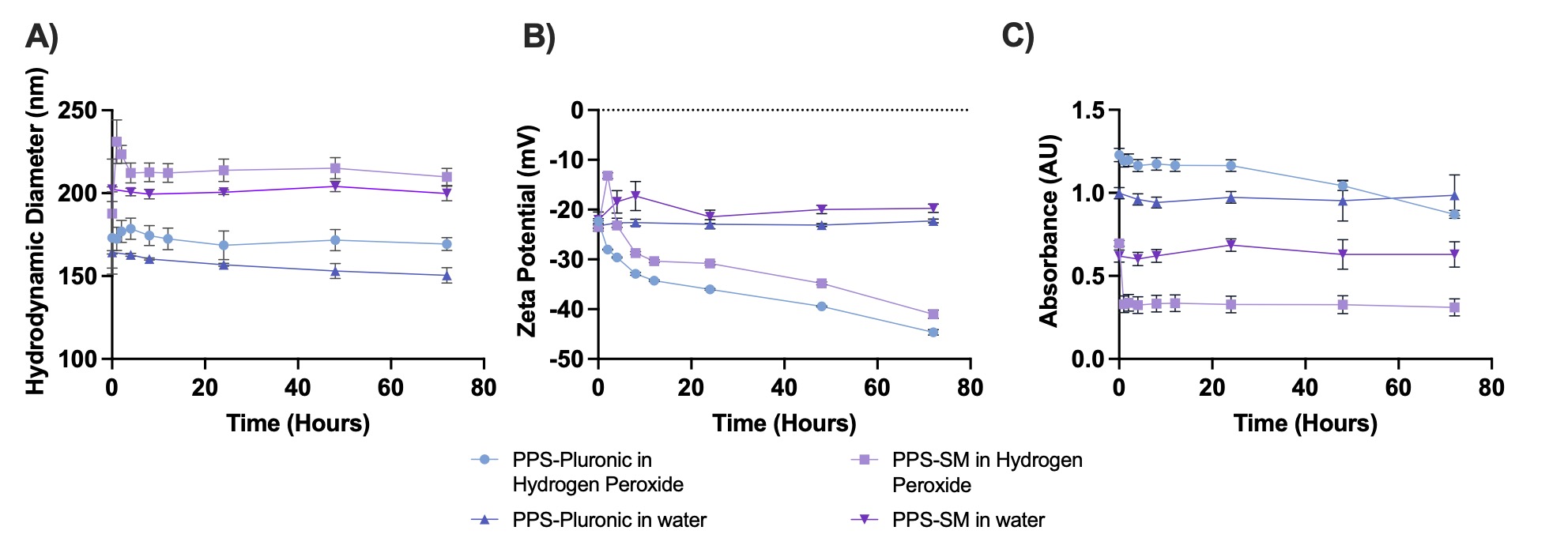


**Figure S2. PPS nanoparticle formulations incubated with 5% hydrogen peroxide or ultrapure water at 25 °C over 72 hours.** A) Hydrodynamic diameter of the nanoparticles. B) Zeta potential of the nanoparticles. C) Turbidity measured as a function of optical density. Data reported as mean (n=9) ± standard error of the mean.

**Figure S3. Pro-inflammatory cytokine release from bone marrow-derived macrophages.** Cells were either untreated, or treated with PPS-Pluronic nanoparticles, PPS-SM nanoparticles, or lipopolysaccharide (LPS) for 3 hours. A) IL-6 release, *p* < 0.01 between nanoparticle formulations. B) MCP-1 release, *p* >0.05 between nanoparticle formulations. C) TNF-alpha release, *p* < 0.001 between nanoparticle formulations. Data are represented as mean (n=6) ± standard error of the mean.

**References**

1. Rehor A, Tirelli N, Hubbell JA. A new living emulsion polymerization mechanism: Episulfide anionic polymerization. *Macromolecules*. 2002;35(23). doi:10.1021/ma0211378
2. Rehor A, Hubbell JA, Tirelli N. Oxidation-sensitive polymeric nanoparticles. *Langmuir*. 2005;21(1). doi:10.1021/la0478043
